# Gap-induced reductions of evoked potentials in the auditory cortex: A possible objective marker for the presence of tinnitus in animals

**DOI:** 10.1016/j.brainres.2017.11.026

**Published:** 2018-01-15

**Authors:** Joel I. Berger, William Owen, Caroline A. Wilson, Adam Hockley, Ben Coomber, Alan R. Palmer, Mark N. Wallace

**Affiliations:** Medical Research Council Institute of Hearing Research, School of Medicine, The University of Nottingham, University Park, Nottingham NG7 2RD, UK

**Keywords:** Tinnitus, Auditory cortex, Noise exposure, Chronic recording

## Abstract

Animal models of tinnitus are essential for determining the underlying mechanisms and testing pharmacotherapies. However, there is doubt over the validity of current behavioural methods for detecting tinnitus. Here, we applied a stimulus paradigm widely used in a behavioural test (gap-induced inhibition of the acoustic startle reflex GPIAS) whilst recording from the auditory cortex, and showed neural response changes that mirror those found in the behavioural tests. We implanted guinea pigs (GPs) with electrocorticographic (ECoG) arrays and recorded baseline auditory cortical responses to a startling stimulus. When a gap was inserted in otherwise continuous background noise prior to the startling stimulus, there was a clear reduction in the subsequent evoked response (termed gap-induced reductions in evoked potentials; GIREP), suggestive of a neural analogue of the GPIAS test. We then unilaterally exposed guinea pigs to narrowband noise (left ear; 8–10 kHz; 1 h) at one of two different sound levels – either 105 dB SPL or 120 dB SPL – and recorded the same responses seven-to-ten weeks following the noise exposure. Significant deficits in GIREP were observed for all areas of the auditory cortex (AC) in the 120 dB-exposed GPs, but not in the 105 dB-exposed GPs. These deficits could not simply be accounted for by changes in response amplitudes. Furthermore, in the contralateral (right) caudal AC we observed a significant increase in evoked potential amplitudes across narrowband background frequencies in both 105 dB and 120 dB-exposed GPs. Taken in the context of the large body of literature that has used the behavioural test as a demonstration of the presence of tinnitus, these results are suggestive of objective neural correlates of the presence of noise-induced tinnitus and hyperacusis.

## Highlights

• Gap-suppression of startle responses is regularly used as a measure for tinnitus.
• We studied this phenomenon in auditory cortical evoked potentials in awake animals.
• Gap-suppression of evoked potentials was also examined following noise exposure.
• 120 dB SPL noise exposure, but not 105 dB, resulted in deficits in gap-suppression.
• Results are discussed in the context of a potential correlate of tinnitus.

## 1 Introduction

Tinnitus, the perception of sound in the absence of an external acoustic stimulus, is a widespread health concern, affecting between 8 and 15% of the population and is debilitating in ∼1% (Shargorodsky et al., 2010). Animal models of the condition are essential for examining the underlying causes and developing potential treatments, and objective assessment of tinnitus is an essential prerequisite (von der Behrens, 2014). In animal models, tinnitus is generally identified by using a behavioural task, either reflex-based (Turner et al., 2006, Berger et al., 2013) or using a conditioning paradigm (Jastreboff et al., 1988, Bauer et al., 1999, Heffner and Harrington, 2002, Lobarinas et al., 2004, Stolzberg et al., 2013). In recent years, reflex-based behavioural tasks have become more common, as they don’t require lengthy training protocols and allow for the examination of tinnitus over a longer period of time than tasks such as lick suppression procedures, due to not involving extinction of behaviour.

The reflex-based test most often used is known as the gap pre-pulse inhibition of the acoustic startle (GPIAS) test, originally developed by Turner et al. (2006). A startling pulse (usually a short broadband stimulus) is embedded in continuous narrowband or broadband noise. This produces a whole-body startle response, which can be detected using a platform with a piezo-electric transducer attached (or motion tracking cameras in the case of the Preyer reflex adaptation; Berger et al., 2013, Wu et al., 2016). When a short gap in the background noise is inserted prior to the startling stimulus, the subsequent startle response is inhibited. If animals are subjected to interventions known to cause tinnitus in both humans and animals - either salicylate administration (Samlan et al., 2008, Stolzberg et al., 2012) or noise exposure (Heffner and Harrington, 2002, Muhr and Rosenhall, 2010) - less inhibition of the response occurs, suggesting that the brainstem mechanisms responsible for inhibiting the startle response have become less effective. The original hypothesis was that tinnitus perceptually filled in the gap and GPIAS deficits are interpreted as objective evidence of tinnitus.

However, there has been some doubt cast over the validity of the GPIAS test in assessing tinnitus (see Galazyuk and Hebert, 2015 for a review). Furthermore, following noise exposure, significant reductions in startle amplitudes may occur, which can result in a false positive assessment of tinnitus as a result of reducing the dynamic range of the response (Lobarinas et al., 2013). Despite potential workarounds to avoid possible pitfalls of the test (e.g. Longenecker and Galazyuk, 2012, Lobarinas et al., 2015), a more direct neural marker of tinnitus in animals would be a useful addition to the pre-existing tests. Perhaps more importantly, the reflex-based tests are likely mediated by brainstem circuits which may not directly involve the auditory cortex (Li and Frost, 1996, Gomez-Nieto et al., 2014). If auditory cortex is essential for the perception of tinnitus, behavioural deficits in a reflex-based task which does not require cortical activation may not directly reflect changes in cortical activity relating to the presence of tinnitus (Eggermont, 2013), although it should be noted that at commonly used gap durations (<50 ms) behavioural performance is still subject to descending cortical modulation (Ison et al., 1991, Threlkeld et al., 2008).

We previously demonstrated that gaps in otherwise continuous background noise could inhibit cortical evoked responses to a startling stimulus in guinea pigs, in a similar manner to the GPIAS test (Berger et al., 2017), which we termed gap-induced reductions in evoked potentials (GIREP). Furthermore, following the induction of tinnitus by administration of sodium salicylate, GIREP was reduced in the same way as the reduction in GPIAS. This was not associated with any changes in the thresholds for detecting the silent gap, so was not simply a result of deterioration in temporal acuity.

Here, we sought to determine whether we could observe deficits in GIREP following noise exposure, which would potentially provide an objective measure of noise-induced tinnitus. To this end, we chronically implanted two groups of guinea pigs (GPs) with electrocorticographic arrays. Following baseline data collection, both groups received the same frequency and duration of noise exposure (8–10 kHz; 1 h; left ear only), but one group was exposed to 105 dB SPL RMS whilst the other received 120 dB SPL RMS. We have previously demonstrated behavioural evidence of tinnitus in guinea pigs using a similar 120 dB paradigm (Coomber et al., 2014). Noise exposure is the most common inducer of tinnitus (Axelsson and Prasher, 2000) and its prevalence is related to the intensity of noise exposure (Turner and Larsen, 2016). We therefore hypothesised that we would observe deficits in GIREP ratios consistent with the presence of tinnitus in the higher level sound exposure group but not the lower exposure level group. We also assessed evoked potential (EP) amplitudes in response to stimuli without a gap preceding, to determine whether these could potentially account for any changes in GIREP observed following noise exposure.

## 2 Results

**Fig. 1:**
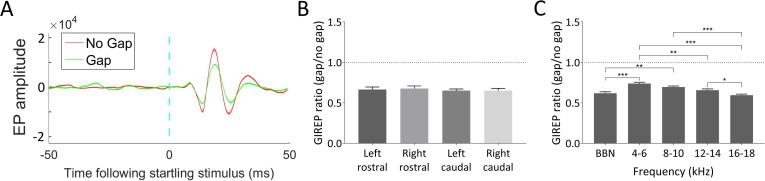
A: An example of GIREP from one GP. Dashed blue line indicates time of startling stimulus. B: Mean (±SEM) GIREP ratios expressed as gap/no gap, for all GPs pre-NE as a function of electrode location. A value of 1 would indicate no difference in EP amplitude between gap and no gap, whilst a value lower than 1 would indicate that the gap was inhibiting the subsequent EP. C: Mean (±SEM) GIREP ratios for all GPs pre-NE as a function of background carrier frequency. ^*^p < .05; ^**^p < .01; ^***^p < .0001.

Fig. 1A shows an example of GIREP for one GP prior to noise exposure, in right caudal AC with a 12–14 kHz background carrier. A clear reduction in the amplitude of the evoked potential in the trials with a gap was evident within a single session. GIREP was calculated as a gap/no gap ratio, whereby a value of 1 would indicate no change in EP amplitude when a gap was presented prior to the startling stimulus, whilst a value lower than 1 would indicate that a preceding gap was reducing the subsequent EP. GIREP was evident for every electrode for all GPs during recordings made before the noise exposure. The mean (±SEM) GIREP ratio across electrodes and GPs before noise exposure (pre-NE) was 0.619 (±0.018) for BBN, 0.739 (±0.015) for 4–6 kHz, 0.696 (±0.012) for 8–10 kHz, 0.657 (±0.016) for 12–14 kHz and 0.595 (±0.013) for 16–18 kHz.

In order to determine whether there was a difference in GIREP between auditory cortical areas or hemispheres, a two-way ANOVA with a Bonferroni *post hoc* test was used to compare GIREP ratios between all four electrodes for each of the five frequencies. There was no significant difference overall between electrode locations (F_(3,515)_ = 0.89, *p* = .56; Fig. 1B), and no interaction between frequency and electrode location (F_(12,515)_ = 0.88, *p* = .45). Although there was a significant overall effect of frequency (F_(4,515)_ = 13.56, *p* < .0001), there were no significant differences between any of the electrodes at any frequency measured. Further analysis revealed that the overall effect of frequency across electrodes was due to lower GIREP ratios at higher frequencies compared to lower frequencies (Fig. 1C), with a significant difference between 4–6 and 12–14 kHz (mean difference in ratio = 0.08; *p* = .001), 4–6 and 16–18 kHz (mean difference in ratio = 0.14; *p* < .0001), 8–10 and 16–18 kHz (mean difference in ratio = 0.10; *p* < .0001), 12–14 and 16–18 kHz (mean difference in ratio = 0.06; *p* = .03), BBN and 4–6 kHz (mean difference in ratio = 0.12; *p* < .0001), and BBN and 8–10 kHz (mean difference in ratio = 0.08; *p* = .003).

**Fig. 2:**
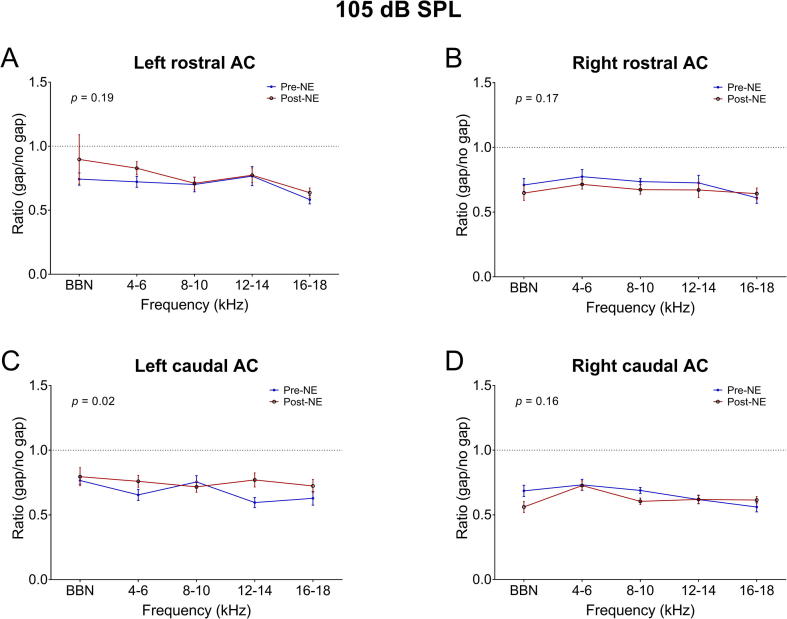
Mean (±SEM) GIREP ratios for GPs exposed to 105 dB SPL (*n* = 5), averaged over pre-NE recordings compared with post-NE averages, for left rostral AC (A), right rostral AC (B), left caudal AC (C) and right caudal AC (D). P-values on subplots indicate overall statistical differences between time points.

GIREP was recorded 7–10 weeks following either 105 dB SPL or 120 dB SPL noise exposure. Two-way ANOVAs were performed for each set of animals at each electrode location, whilst differences at each frequency were examined with Bonferroni *post hoc* tests. Across the GPs exposed to 105 dB SPL, although there was a significant overall effect of time point for left caudal AC (F_(1,110)_ = 5.679, *p* = .02), and a significant overall difference between frequencies for right caudal AC (F_(4,152)_ = 4.66, *p* < .001), there were no significant changes in GIREP at any particular frequencies for any of the cortical areas (Fig. 2).

**Fig. 3:**
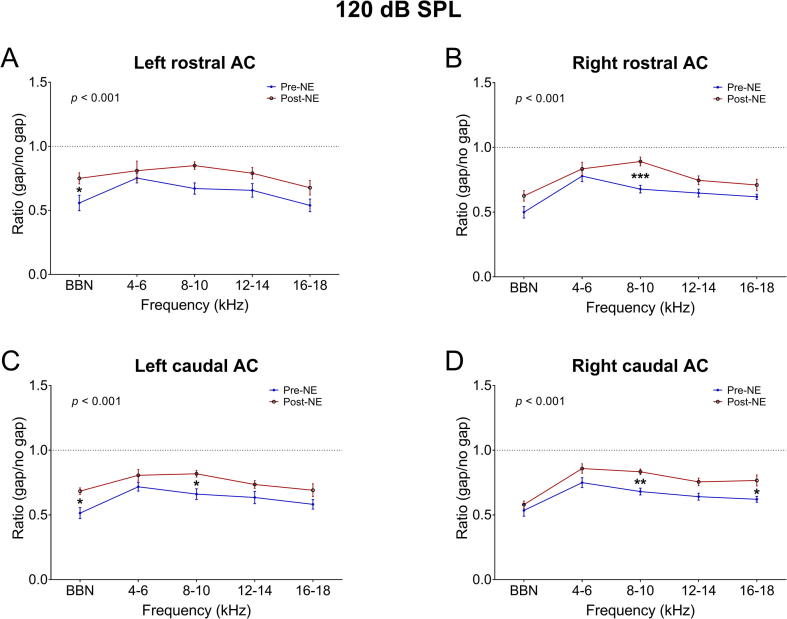
Mean (±SEM) GIREP ratios for GPs exposed to 120 dB SPL (*n* = 4), averaged over pre-NE recordings compared with post-NE averages, for left rostral AC (A), right rostral AC (B), left caudal AC (C) and right caudal AC (D). ^*^p < .05; ^**^p < .01; ^***^p < .0001. P-values on subplots indicate overall statistical differences between time points.

Fig. 3 shows GIREP data for GPs exposed to 120 dB SPL. There were overall effects of time point and frequency for left caudal AC (F_(1,105)_ = 18.85, *p* < .001 and F_(4,105)_ = 4.077, *p* = .004, respectively), right caudal AC (F_(1,145)_ = 23.33, *p* < .001 and F_(4,145)_ = 13.21, *p* < .001), left rostral AC (F_(1,105)_ = 25.51, *p* < .001 and F_(4,105)_ = 6.09, *p* < .001), and right rostral AC (F_(1,145)_ = 30.17, *p* < .001 and F_(4,145)_ = 16.18, *p* < .001). Contrary to the 105 dB GPs, Bonferroni *post hoc* tests revealed that 120 dB SPL-exposed GPs exhibited GIREP deficits after noise exposure (post-NE) at 8–10 kHz in right rostral AC (*p* = .0006), left caudal AC (*p* = .03), and right caudal AC (*p* = .006). There was also a smaller yet significant deficit at 16–18 kHz in the right caudal AC (*p* = .01), and at BBN in left rostral AC (*p* = .04) and left caudal AC (*p* = .01). GIREP at other frequencies in all cortical areas were reduced but did not reach significance.

**Fig. 4:**
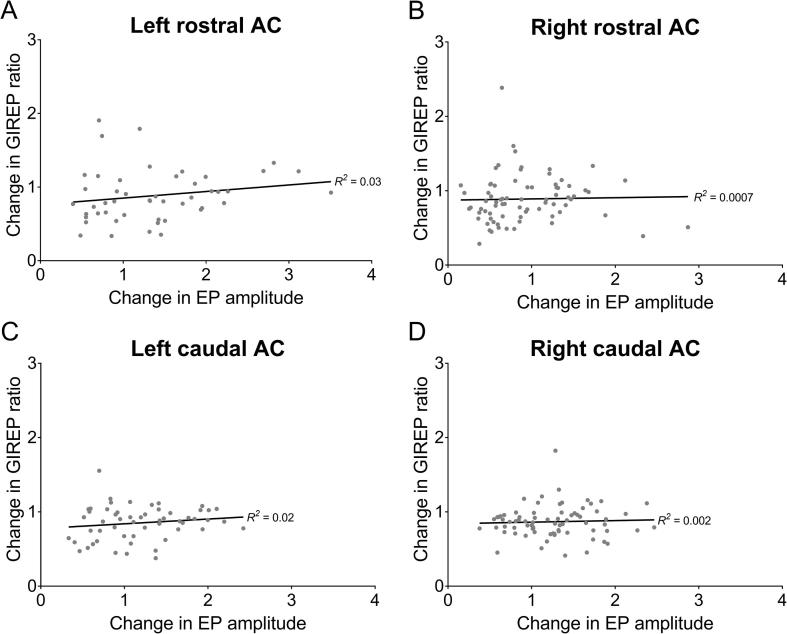
Correlations between changes in GIREP ratios and changes in EP amplitude (in response to stimuli with no gap preceding) for left rostral AC (A), right rostral AC (B), left caudal AC (C) and right caudal AC (D), recorded from 120 dB GPs. Data have been fitted with linear regressions to demonstrate the lack of significant trends (solid black lines).

In order to determine whether deficits in GIREP in the 120 dB GPs could be accounted for by decreases in EP amplitudes, peak-to-trough amplitudes in response to the startling stimulus with no gap preceding were assessed for each frequency, comparing pre-NE to post-NE. This was important to examine, as decreased EP amplitudes in response to the startling stimulus may have related to hearing loss caused by the noise exposure, which would have reduced the dynamic range of the EP (and therefore potentially decrease GIREP ratios). This would reflect a similar issue observed in the GPIAS test, wherein reduced startle responses following noise exposure may result in false positive identification of tinnitus (Lobarinas et al., 2013). However, the only significant decrease in EP amplitude caused by noise exposure was at BBN in right rostral AC (*t*(145) = 3.59, *p* = .002), and in many cases EP amplitudes were actually increased, albeit not to a significant extent at any one frequency. Spearman’s Rho tests showed that there was no significant correlation between changes in EP amplitudes and changes in GIREP ratios (calculated as a before vs after ratio; Fig. 4) for the left rostral AC (R_s_ = 0.27, *p* = .07), left caudal AC (R_s_ = 0.14, *p* = .32), right rostral AC (R_s_ = 0.20, *p* = .09) or right caudal AC (R_s_ = 0.05, *p* = .64).

**Fig. 5:**
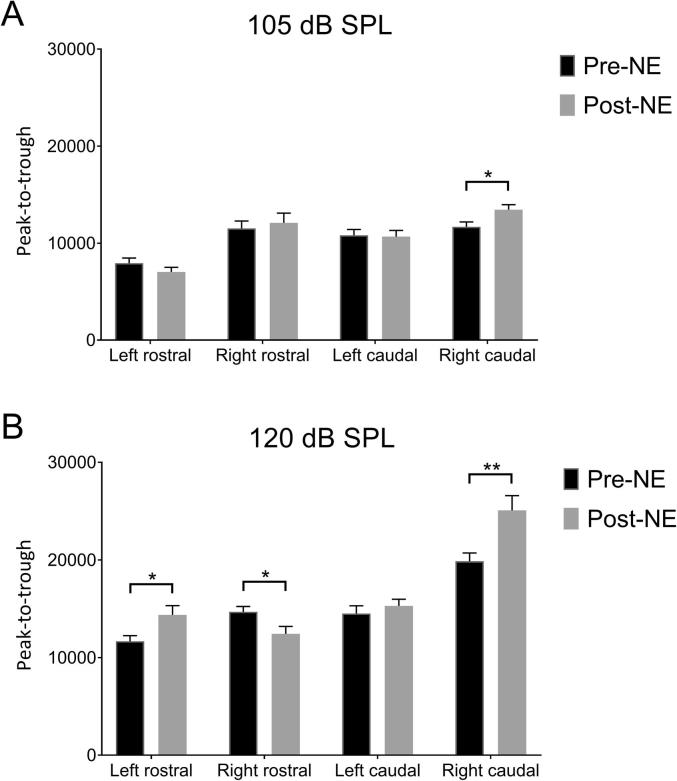
Comparisons of peak-to-trough amplitudes, averaged across frequencies, for each area of AC. Mean (±SEM) data are shown for 105 dB SPL GPs (A) and 120 dB SPL (B), for pre-NE vs post-NE. ^*^p < .05; ^**^p < .01.

Fig. 5 shows EP amplitudes, for each area of AC, for 105 dB (Fig. 5A) and 120 dB (Fig. 5B) GPs. When averaging across all frequencies (Fig. 5B), a significant increase in EP amplitudes post-NE for the 120 dB GPs was evident for left rostral AC (*t*(54) = 2.61, *p* = .01) and right caudal AC (*t*(74) = 3.51, *p* = .0008), whilst amplitudes in right rostral AC decreased (*t*(74) = 2.53, *p* = .01) and there was no significant change in left caudal AC (*t*(54) = 0.58, *p* = .56). For the 105 dB GPs, there was a slight yet significant increase in the right caudal AC (*t*(74) = 2.05, *p* = .04), whilst amplitudes in the other cortical areas did not significantly change (Fig. 5A).

To further explore these data, we also performed two-way ANOVAs, using frequency and time point as the independent variables. The only main effect of noise exposure for the 105 dB GPs was in the right caudal AC (F_(1,152)_ = 6.39, *p* = .01), supporting the results of the corrected *t*-tests, with no significant main effect of frequency (F_(4,152)_ = 2.41, *p* = .05) nor any significant *post hoc* differences. For the 120 dB GPs, main effects of noise exposure were evident for left rostral AC (F_(1,105)_ = 5.80, *p* = .02), right rostral AC (F_(1,145)_ = 7.07, *p* = .008) and right caudal AC (F_(1,145)_ = 9.96, *p* = .002), again supporting the results of the corrected *t*-tests. Effects of frequency were evident for left rostral AC (F_(4,105)_ = 3.63, *p* = .008), right rostral (F_(4,145)_ = 6.71, *p* < .001), left caudal AC (F_(4,105)_ = 4.07, *p* = .004), and right caudal AC (F_(4,145)_ = 9.96, *p* = .002). A slight yet significant interaction effect (between frequency and time point) was evident in the left caudal AC (F_(4,105)_ = 2.74, *p* = .03) and the only significant *post hoc* difference was a reduction in EP amplitudes at BBN in the right rostral AC (*p* = .002), which was again consistent with the results of the *t*-tests. In summary, these data suggest that whilst there was a general increase in EP amplitudes at a number of electrode locations, this was not isolated to any particular background frequency.

## 3 Discussion

The current study assessed whether different noise exposure levels would induce varying changes in neural responses consistent with the presence of tinnitus. We were successfully able to identify deficits in GIREP in the contralateral AC using 120 dB SPL noise exposure, particularly at the noise exposure frequency, but not with 105 dB SPL. Smaller deficits were also evident in the ipsilateral AC, albeit not to the same degree. All of these deficits could not be accounted for simply by reductions in EP amplitudes, which were in fact increased in many cases. Based on previous studies using GPIAS as a behavioural test for tinnitus in animals, these data are suggestive of objective neural changes associated with noise-induced tinnitus and hyperacusis, and highlight a dependence on the noise exposure sound level used.

Crucially, during pre-NE recordings, we were able to demonstrate that we could successfully inhibit an EP response to a startling stimulus by inserting a preceding gap in continuous background noise (i.e. GIREP) for all background carrier frequencies. This was consistent with Berger et al. (2017), wherein we observed this phenomenon for the first time. Whilst these are the first instances of gap-induced suppression of cortical evoked responses in animals, a similar phenomenon was observed in humans by Ku et al. (2015). They demonstrated successful gap-induced inhibition of the auditory late response following an intense sound burst in healthy volunteers, measured using electroencephalography. In a recent follow-up study, Ku et al. (2017) found deficits in gap-induced suppression of auditory late responses in tinnitus patients, present at the frequency that best matched their tinnitus, supporting the idea that this may be an objective neural measure of tinnitus, though some deficits were also observed at a much lower frequency, albeit in both patients and controls.

Interestingly, GIREP ratios recorded pre-NE were inversely related to the background carrier frequency, meaning that a gap embedded in a higher frequency carrier was more effective at reducing a subsequent EP than one embedded in a low frequency carrier. This is somewhat surprising given that the most sensitive frequency of the guinea pig audiogram is ∼8–10 kHz (Heffner et al., 1971, Prosen et al., 1978), which is the second lowest frequency we measured, therefore suggesting that GIREP is more effective at frequencies above this. To our knowledge, there is no published data on the frequency dependency of gap-induced reductions of either startle or neural response in guinea pigs. There are data in other rodents indicating that GPIAS is greater at higher frequencies compared to lower frequencies, although this was only evident in rats (Steube et al., 2016) and mice (Ison et al., 2005), wherein the most sensitive part of the audiogram is at higher frequencies than guinea pigs. This effect was not evident in gerbils (Kiefer et al., 2015, Steube et al., 2016) and another study failed to demonstrate frequency-dependency of GPIAS in rats (Lobarinas et al., 2015). As Steube et al. (2016) stated, it is possible that the frequency-dependency of GPIAS is different between species. Furthermore, as we used the same linear bandwidths for all background carrier frequencies, the higher frequency carriers would occupy narrower areas on the basilar membrane (due to its nonlinearity). Therefore, it is plausible that the higher frequencies could be more sharply tuned in cortex and produced a more sharply focussed inhibition. Clearly, more work is required to understand the interaction between background carrier frequency and extent of reductions in startle response amplitudes, both in behavioural and neural data, in a variety of species.

Following 120 dB SPL noise exposure, the clearest deficits in GIREP observed here were at the noise exposure frequency (8–10 kHz) on the contralateral side. The precise mechanisms of the original GPIAS behavioural test have been disputed, and given the current data we could only speculate as to the mechanisms behind GIREP deficits. The original hypothesis was that tinnitus was perceptually filling in the gap (Turner et al., 2006), although others contend that deficits may instead reflect problems with temporal processing (Campolo et al., 2013, Fournier and Hebert, 2013). Whilst the current study does not resolve this issue, by recording directly from auditory cortex it does overcome the caveat posed by Eggermont (2013): that is, animal behavioural tests which are mediated by brainstem circuitry (such as the GPIAS test) may only reflect subcortical changes following an intervention and, therefore, if the auditory cortex is essential for tinnitus perception, may not accurately reflect the perception of tinnitus. The data presented here suggest that deficits in GIREP do reflect changes in auditory cortex induced by the noise exposure, which cannot simply be accounted for by reduced activity in that area (as EP amplitudes were not consistently decreased in the presence of GIREP deficits) and therefore may be interpreted as the presence of tinnitus. Furthermore, the fact that EP amplitudes did not generally decrease means that it also avoids the confounding factor of false positive identification of tinnitus as a result of reduced startle amplitudes (Lobarinas et al., 2013).

Although studies in humans have demonstrated that patients with tinnitus can still perform psychophysical gap detection tasks as well as controls (Campolo et al., 2013, Boyen et al., 2015), as we previously discussed (Berger et al., 2017), it is likely that there are fundamental differences between gap-induced reductions of a reflex response (or, in this case, EPs) and absolute gap detection thresholds. We previously demonstrated that GIREP deficits were present following salicylate administration in guinea pigs, in the absence of changes in either gap termination responses or minimum gap detection thresholds (MGDTs). We have also demonstrated that deficits in MGDTs following noise exposure were not sufficient to explain behavioural gap detection deficits (Berger et al., 2014). Furthermore, Weible et al. (2014) demonstrated important differences between the GPIAS test and MGDTs using optogenetics in mice to alter cortical inhibition or excitation, demonstrating that they could affect the degree of startle attenuation in the behavioural task whilst preserving MGDTs. Therefore, the current measure of GIREP may well be a measure of gap salience, rather than temporal acuity. If the presence of tinnitus is assumed to cause deficits in gap salience, then this may be a more appropriate measure to use than absolute gap detection thresholds.

It was interesting that both groups of noise-exposed GPs had significant increases in their EP amplitudes in right caudal AC. A number of previous animal studies have demonstrated increases in cortical EP amplitudes following noise exposure, which have often been attributed to hyperacusis (Popelar et al., 1987, Syka et al., 1994, Syka and Rybalko, 2000, Sun et al., 2008, Sun et al., 2012). Hyperacusis is characterised by an oversensitivity to sound and is often co-morbid with tinnitus, suggesting that the two may have similar underlying aetiologies (e.g. Anari et al., 1999, Dauman and Bouscau-Faure, 2005). However, in the 105 dB GPs, these increases were evident in the absence of evidence of GIREP deficits, a possible indicator of tinnitus. If increases in EP amplitudes are assumed to be a correlate of hyperacusis, this therefore suggests two points: (1) that hyperacusis can occur without the presence of tinnitus, something which, although rarer, is sometimes reported in the human literature (Jastreboff and Hazell, 1993, Moliner Peiro et al., 2009, Meuer and Hiller, 2015) and (2) hyperacusis may be induced at lower noise exposure levels than tinnitus. However, whilst we demonstrate GIREP deficits that may relate to tinnitus, it is important to note that there is no certainty that the tinnitus percept itself underlies these effects and, furthermore, there is controversy over whether animals may experience tinnitus or hyperacusis in the same way that humans do (Eggermont, 2013). Therefore, clearly further work is required to fully understand the differences in mechanisms between tinnitus and hyperacusis.

The GIREP test would not necessarily be an appropriate replacement for the routine behavioural verification of tinnitus in animals. The expense of the equipment and time-consuming nature of surgically-implanting every animal is above that of a standard GPIAS behavioural set up. However, if animals are already being implanted with electrodes then the GIREP method would be a useful test to implement to identify the presence of tinnitus. Ideally, although a group analysis was performed here (due to limited GIREP data under certain circumstances), enough sessions would be collected to reliably perform statistical analysis on individual animals, as it is likely that not all animals would have developed tinnitus following the same noise exposure (Turner and Larsen, 2016). The GIREP test could also act as a complement to measuring animal behavioural performance and would be a useful tool to examine whether various treatments could abolish deficits in GIREP consistent with the presence of tinnitus. Whilst we did not have behavioural data to corroborate the deficits we observed in GIREP in the current study, we demonstrated previously that salicylate-induced deficits in GIREP were closely aligned with behavioural deficits in guinea pigs (Berger et al., 2017). Moreover, GIREP may have utility in confirming evidence of tinnitus where shortcomings in the behavioural test, such as significant reductions in startle amplitudes following intense noise exposure, are present. Additionally, by recording directly from the auditory cortex, any deficits are likely to reflect changes in cortical activity. If auditory cortex changes underlie the presence of tinnitus, this is an important caveat that is overcome by utilising the GIREP test.

## 4 Methods and materials

### 4.1 Animals

All procedures were carried out in accordance with the European Communities Council Directive of 24 November 1986 (86/609/EEC) and the approval of the Animal Welfare and Ethical Review Body at the University of Nottingham, UK. Experiments were conducted on a total of 9 male and female guinea pigs, weighing between 860 and 1180 g at the time of noise exposure.

### 4.2 Electrocorticography (ECoG) array implantation

**Fig. 6:**
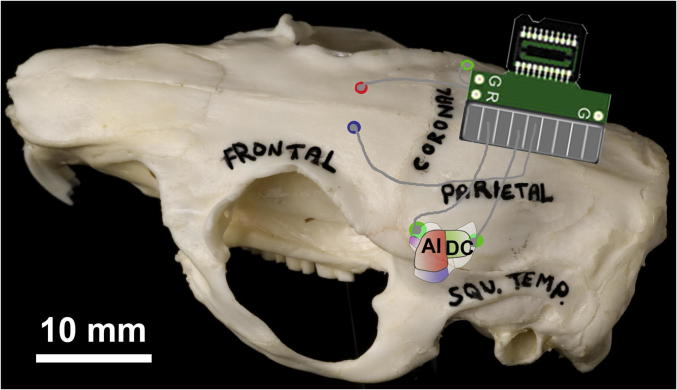
Diagram showing the position of the recording electrodes and connector in relation to a guinea pig skull and the underlying auditory cortical areas as mapped previously (Wallace et al., 2000). The rostral recording electrode was placed over the dorsorostral belt close to the low-frequency border of the primary auditory area (AI, coloured red) whilst the caudal electrode was placed over the dorsocaudal belt close to the low-frequency border of the dorsocaudal core area (DC, coloured green). The lateral suture, which forms the border of the parietal and squamous temporal bones, provides a useful surface landmark as it runs approximately over the middle of AI before turning towards the midline and forming the coronal suture. Red and blue electrodes served as reference and ground.

The methodology for ECoG array preparation and implantation is described in detail in Berger et al. (2017). Briefly, uninsulated silver wires with silver ball tips were soldered onto a printed circuit board attached to a Tucker Davis Technologies (TDT: Alachua, FL, USA) zero insertion force (ZIF)-clip connector. GPs were initially anaesthetised on a mixture of ketamine (40 mg/kg, i.p.; Fort Dodge Animal Health Ltd, Southampton, UK) and xylazine (8 mg/kg, i.p.; Bayer PLC, Newbury, UK) and maintained on an isoflurane/O_2_ mixture. Following a midline incision, muscle and connective tissue were cleared, and electrodes were placed on the surface of the dura through small burr holes in the skull. Auditory cortex (AC) electrodes were positioned approximately 1 mm (rostral AC) and 5 mm (caudal AC) behind bregma, close to the lateral suture on either side (See Fig. 6). Reference and ground electrodes were linked on the electrode board and implanted ∼3 mm rostral to bregma. Two small stainless steel screws were also inserted into burr holes to help anchor the assembly to the skull. The underside of the board and the electrode holes were coated with Kwik-Cast silicone (World Precision Instruments: Hitchin, UK) and covered with dental acrylic. The wound was sutured with Mersilk (Ethicon: Livingston, UK). Antibiotic cream and cyanoacrylate adhesive were applied to the wound surrounding the board, and GPs were left to recover for at least 24 h prior to baseline ECoG recording.

### 4.3 Recording setup

Chronic ECoG recordings were made inside a sound-attenuated chamber, with a ZIF-clip headstage attached to the implanted electrode. GPs were awake and freely moving throughout recording. Auditory stimuli were presented free-field via a single ¾-inch tweeter (Tymphany XT19TD00) positioned ∼30 cm above the centre of the cage. Two ¼-inch free-field microphones attached to a preamplifier (G.R.A.S. 40BP and 26AC, respectively), placed at either end of the cage, were used to calibrate signals. Recorded ECoG signals were filtered online between 60 and 300 Hz. Data collection was facilitated by Brainware (J. Schnupp, University of Oxford, UK). Custom-written Matlab scripts (R2014b, MathWorks, MA, USA) were used for offline analysis. Only data from fully functional electrodes were included in analysis.

### 4.4 GIREP stimuli

Cortical evoked responses were recorded to startling stimuli (broadband noise bursts of 20 ms duration; rise/fall time of 1 ms) embedded in five different continuous background noise conditions (either BBN or 2 kHz wide narrow-band noise (NBN) centred at 5, 9, 13, or 17 kHz), in the same manner as described previously (Berger et al., 2017). Gaps of 50 ms duration, starting 100 ms before the startling stimulus, were randomly inserted on half of the trials, resulting in 10 gap/no gap conditions for each frequency. Sound levels were determined behaviourally prior to implantation for each GP as described in our previous work, with startling stimuli of either 95, 100, or 105 dB SPL and background carrier stimuli of 55, 60, or 70 dB SPL (sound level-dependency test; see Berger et al., 2013). EP amplitudes were determined using peak-to-trough amplitudes of ECoG signals in the 50 ms following the startling stimulus, averaged across repeats. Data were collected for a minimum of three separate sessions at each time point.

### 4.5 Noise exposure

Following baseline GIREP data collection, each GP was exposed to loud noise. Anaesthesia was induced with ketamine (50 mg/kg, i.p.) and xylazine (10 mg/kg, i.p.) and maintained with a 15:2 ratio mixture of ketamine and xylazine. Core body temperature was monitored and maintained at 38 ± 0.5 °C with a homeothermic heating pad (Harvard Apparatus Ltd., Edenbridge, UK) attached to a rectal probe. For 105 dB SPL exposed GPs, a TDT MF1 speaker with a 35 mm long PVC tube attached (1.67 mm inner diameter) was used to present sound to the left ear, positioned in the ear canal and surrounded near the tip with petroleum jelly to create a seal. For 120 dB SPL exposed GPs, a 25 mm loudspeaker (Peerless DX25, Tymphany, Hong Kong) was used with a 20 mm diameter polyethylene tube surrounding the left pinna, as this speaker was capable of producing louder stimuli than the TDT MF1. Levels were calibrated to either 105 dB SPL or 120 dB SPL, using a 40BP ¼-inch microphone connected to a 26AC preamplifier (both G.R.A.S.) with a calibrated 1-mm-diameter probe attached. In both cases, unilateral (left-ear) noise exposure stimuli consisted of 8–10 kHz narrowband noise for 1 h. We have previously demonstrated behavioural evidence of tinnitus following 120 dB SPL noise exposure for 1 h (Coomber et al., 2014), whilst 105 dB noise exposure for 1 h is currently considered as the limit before sound levels become traumatising (Gourevitch et al., 2014). The right pinna was folded and surrounded with a polyethylene tube plugged with cotton wool, in order to ensure that only the left ear was exposed. GPs remained inside a sound-attenuating booth for the duration of the noise exposure. Post-noise exposure GIREP recordings were performed 7–10 weeks following the procedure.

### 4.6 Data analysis

To quantify the amount of GIREP, differences in EP amplitudes between ‘no gap’ and ‘gap’ conditions were calculated to give gap/no gap ratios for each electrode. For both groups of noise-exposed GPs (105 and 120 dB SPL), comparisons were made between evoked potentials recorded before noise exposure (‘pre-NE’) compared with evoked potentials recorded 7–10 weeks following noise exposure (‘post-NE’). We have previously demonstrated tinnitus-like behaviour at this time point (Berger et al., 2014, Coomber et al., 2014), and it has been suggested that neural changes caused by noise exposure become centralised by 8 weeks following the exposure (Mulders and Robertson, 2011). The recording session with the lowest GIREP for each GP from each time point was discarded prior to analysis to prevent skewing of the data. These time points were then statistically compared for each electrode across all GPs and all frequencies using two-way ANOVAs with Bonferroni *post hoc* tests. In order to examine whether an overall decrease in amplitudes could account for any GIREP deficits observed, peak-to-trough EP amplitudes in response to stimuli without a gap preceding were assessed using a planned comparison of multiple *t*-tests with Holm-Sidak correction applied. These data were further explored using two-way ANOVAs with Bonferroni *post hoc* tests. Spearman’s rho analyses were used to correlate changes in EP amplitudes (in response to stimuli without a gap preceding) with changes in GIREP ratios.
